# Dietary Vitamins A, C, and Potassium Intake Is Associated With Narrower Retinal Venular Caliber

**DOI:** 10.3389/fmed.2022.818139

**Published:** 2022-02-10

**Authors:** Ayaka Edo, Diah Gemala Ibrahim, Kazuyuki Hirooka, Rie Toda, Muhammad Irfan Kamaruddin, Reo Kawano, Akiko Nagao, Haruya Ohno, Masayasu Yoneda, Yoshiaki Kiuchi

**Affiliations:** ^1^Department of Ophthalmology and Visual Science, Graduate School of Biomedical and Health Sciences, Hiroshima University, Hiroshima, Japan; ^2^Department of Ophthalmology, Hasanuddin University, Makassar, Indonesia; ^3^Clinical Research Center in Hiroshima, Hiroshima University Hospital, Hiroshima, Japan; ^4^Division of Nutrition Management, Hiroshima University Hospital, Hiroshima, Japan; ^5^Department of Molecular and Internal Medicine, Graduate School of Biomedical and Health Sciences, Hiroshima University, Hiroshima, Japan

**Keywords:** retinal vascular caliber, nutrient, vitamin A, vitamin C, potassium

## Abstract

**Introduction:**

The retinal vasculature, a surrogate for the systemic microvasculature, can be observed non-invasively, providing an opportunity to examine the effects of modifiable factors, such as nutrient intake, on microcirculation. We aimed to investigate the possible associations of dietary nutrient intake with the retinal vessel caliber.

**Methods:**

In this cross-sectional study, a total of 584 participants in a medical survey of Japanese descendants living in Los Angeles in 2015 underwent a dietary assessment, fundus photographic examination, and comprehensive physical and blood examinations. Retinal vessel caliber was measured using fundus photographs with a semi-automated computer system and summarized as central retinal artery and vein equivalents (CRAE and CRVE). The association between dietary nutrient intake and retinal vessel caliber was analyzed using a multivariate linear regression model adjusted for two models including potential confounders. The first model was adjusted for age and sex. The second model was adjusted for age, sex, smoking status, body mass index, hypertension, diabetes, dyslipidemia, history of coronary heart disease, and history of stroke.

**Results:**

After adjustment of potential confounders, compared to the quartile with the lowest intake, the difference in CRVE for the highest quartile was −5.33 μm [95% confidence interval (CI): −9.91 to −0.76, *P* for trend = 0.02] for vitamin A, −4.93 μm (95% CI: −9.54 to −0.32, *P* for trend = 0.02) for vitamin C and −3.90 μm (95% CI: −8.48 to 0.69, *P* for trend = 0.04) for potassium.

**Conclusions:**

A significant association was observed between higher vitamins A, C and potassium intakes and narrower retinal venular caliber.

## Introduction

The retinal microvasculature is the easiest and most widely used vascular bed that can be directly visualized *in vivo* and may provide a non-invasive, surrogate method to study early structural changes and pathological features of the human microcirculation (1–3). Over the past two decades, a computerized method of measuring the retinal vascular caliber using retinal photographs has been developed (4). Deviations from the optimal structure of the retinal vasculature have been shown to involve narrower retinal arterial caliber and wider retinal venular caliber, which have been demonstrated to independently predict coronary heart disease (CHD) and stroke (2, 5, 6).

Modificable dietary factors are presumably associated with cardiovascular disease; meta-analyses have shown that higher intake of fish, nuts, fruits, and vegetables is inversely associated with the development of CHD (7–9). It has been reported that dietary nutrition intake of antioxidants, vegetable proteins, potassium, magnesium, and fiber might be partially effective in reducing the risk of CHD and stroke, independent of cardiovascular risk factors (10–14). Similarly, the relationship between retinal microvascular caliber and dietary factors is under investigation. Gopinath et al. (15) have reported that the consumption of a high-quality diet, reflecting high compliance with published dietary guidelines or recommendations, is associated with an advantageous retinal microvascular profile, that is, a wider retinal arteriolar caliber and narrower retinal venular caliber. Keel et al. (16) reported that lower intake of vegetables and fish is associated with wider retinal venular caliber in children and adolescents with type 1 diabetes. Some studies have shown that higher dietary fiber (17), yogurt (18), and fish (19) consumption is associated with a wider retinal arterial caliber and narrower retinal venular caliber. However, these reports are limited and sufficient evidence has not been established.

Since 1970, we have conducted medical surveys of Japanese Americans who migrated from Japan to the United States and their descendants, an epidemiological study called the Hawaii–Los Angeles–Hiroshima Study, to investigate the effects of environmental factors on disease structures among Japanese people (20). We hypothesized that there may be an association between various nutrient intakes and retinal microvascular caliber, just as there is an association between dietary factors and cardiovascular disease. To substantiate this hypothesis, we conducted a study in 2015 using fundus photographic examination and dietary assessment together with a computer-assisted menu suggestion system for Japanese Americans living in Los Angeles, California. The purpose of this study was to investigate the association between retinal vascular caliber and dietary nutritional intake.

## Methods

### Study Design and Population

As part of the Hawaii–Los Angeles–Hiroshima Study (20), medical examinations were conducted in Los Angeles for Japanese Americans in August 2015. The medical examinations were announced through the local Japanese newspaper “Rafu Shimpo” and radio advertisements in Los Angeles; a total of 584 Japanese-Americans participated. First-generation Japanese immigrants from Japan and their descendants born and raised in the United States (second-generation and later) were included. Individuals who had mixed/non-Japanese ethnicity were excluded. All participants received an explanation of the study procedures and provided written informed consent. Participants underwent physical, dietary, and fundus photographic examination by a team of well-trained internists, optometrists, dietitians, and nurses. This study was carried out in accordance with the Declaration of Helsinki and approved by the Ethics Committee of Hiroshima University (No. E-139).

### Dietary Assessment

Dietary intake and dietary habits of all participants were assessed by a food frequency method, as previously described (21–23). First, a paper questionnaire concerning the frequency of food intake was given to the participants. Then, two trained dietitians conducted detailed interviews regarding the frequency of food intake, amount consumed per meal, and preparation method for each food group; they used food models and real foods, while observing the results in personal interviews. They calculated the values for daily total energy and intake for individual nutritional elements [i.e., animal protein, vegetable protein, animal fat, vegetable fat, saturated fatty acids (SFA), polyunsaturated fatty acids (PUFA), cholesterol, carbohydrates, fiber, vitamins A, B1, B2 and C, calcium, iron, potassium and salt]. The average daily intake of each food group was calculated as (average intake per meal) × (frequency of intake per day); the nutrient intake from each food group was calculated as (nutrient value per 1 gram of each food) × (average daily intake of each food group) (21–23). The nutritional value of each food group was determined on the basis of the US Department of Agriculture Nutritive Value of American Foods in Common Units (24).

### Retinal Vascular Caliber Measurement

Bilateral fundus photographs were captured with a 45 degree non-mydriatic retinal camera (NIDEK AFC-300, NIDEK CO., LTD., Gamagori, Japan). Retinal vascular caliber was measured using a semi-automated computer imaging program (Retinal Analysis-IVAN, University of Wisconsin, Madison, WI) by a trained ophthalmologist masked to participants' clinical data (4, 25, 26). Images were presented randomly to a grader. Two circular grids with radii of 0.5 and 1.0 disc diameters were semi-automatically drawn from the edge of the disc; the calibers of all arterioles and venules passing completely through the region of 0.5–1.0 disc diameter were measured. Using the calibers of the six widest arterioles and venules, the central retinal artery and vein equivalents (CRAE and CRVE) were summarized according to the formulae described by Parr-Hubbard (25) and revised by Knudson (27). For the reproducibility of retinal vascular measurements, the intraclass correlation coefficient was high (>0.9). For this study, data of the right eye were included. When the right eye was ungradable, we used data from the left eye.

### Assessment of Covariates

All participants underwent an interview and comprehensive physical examination, and each provided a blood sample after an overnight fast. Venous blood was collected to measure high-density lipoprotein cholesterol (HDL-C), low-density lipoprotein cholesterol (LDL-C), triglyceride (TG) and blood glucose levels. These measurement methods are described elsewhere (28). Height and weight were measured using a digital scale with a stadiometer. Body mass index (BMI) was then calculated as weight divided by height squared (kg/m^2^). Information regarding smoking history and a previous diagnosis of hypertension, diabetes, dyslipidemia, CHD, and stroke was obtained in personal interviews. According to participants' self-reports, smoking history was categorized as never, former, and current smoker. Hypertension was determined as a history of hypertension or using mean arterial blood pressure (MABP), calculated as one-third of the systolic blood pressure plus two-thirds of the diastolic blood pressure; hypertension was defined as MABP ≥105.68 mmHg (29). Each participant without diabetes underwent fasting serum glucose measurement and a 75-g oral glucose tolerance test (OGTT). In line with American Diabetes Association guidelines (30), diabetes was defined as either a previous diabetes diagnosis, a fasting serum glucose level of ≥126 mg/dL or a 2-h serum glucose level of ≥200 mg/dL after an OGTT. Dyslipidemia was defined as a history of dyslipidemia diagnosis, HDL-C <40 mg/dL, LDL-C ≥140 mg/dL, or TG ≥150 mg/dL (31).

### Statistical Analysis

In this study, CRAE and CRVE were examined as continuous dependent variables. Continuous variables are expressed as mean ± standard deviation (SD). First, the mean values of CRAE and CRVE were compared according to participants' background data. After application of the Anderson–Darling test for each variable, the Wilcoxon rank-sum test was used for comparisons between two groups, and the Kruskal–Wallis test was used for comparisons between three or more groups (smoking status and BMI). Next, we used multivariate linear regression model to evaluate the association between retinal vascular caliber (CRAE and CRVE) and dietary nutrient intake. In model 1, we adjusted for age (years, continuous) and sex (male/female). To subsequently assess the effect of confounders, model 2 was additionally adjusted for several known potential confounding factors: smoking status (current/former/never), BMI (kg/m^2^, continuous), hypertension (yes/no), diabetes (yes/no), dyslipidemia (yes/no), history of CHD (yes/no), and history of stroke (yes/no) (1). Spearman's rank correlation test was performed to examine correlations regarding nutrient intake. Each dietary nutrient intake was adjusted for total energy intake using the residual method described by Willett et al. (32) and was categorized into quartiles, with the first quartile indicating lower intake. Differences in retinal vascular caliber per quartile of nutrient intake were estimated using the first quartile as a reference. *P*-values for trend were estimated using nutrient intake as a continuous variable. All statistical analyses were performed using JMP Pro statistical software 15.0.0 (SAS Institute Inc., Cary, NC, USA). All *P*-values were two-sided, and a *P*-value < 0.05 was considered significant.

## Results

### Characteristics of Study Participants

**Figure 1:**
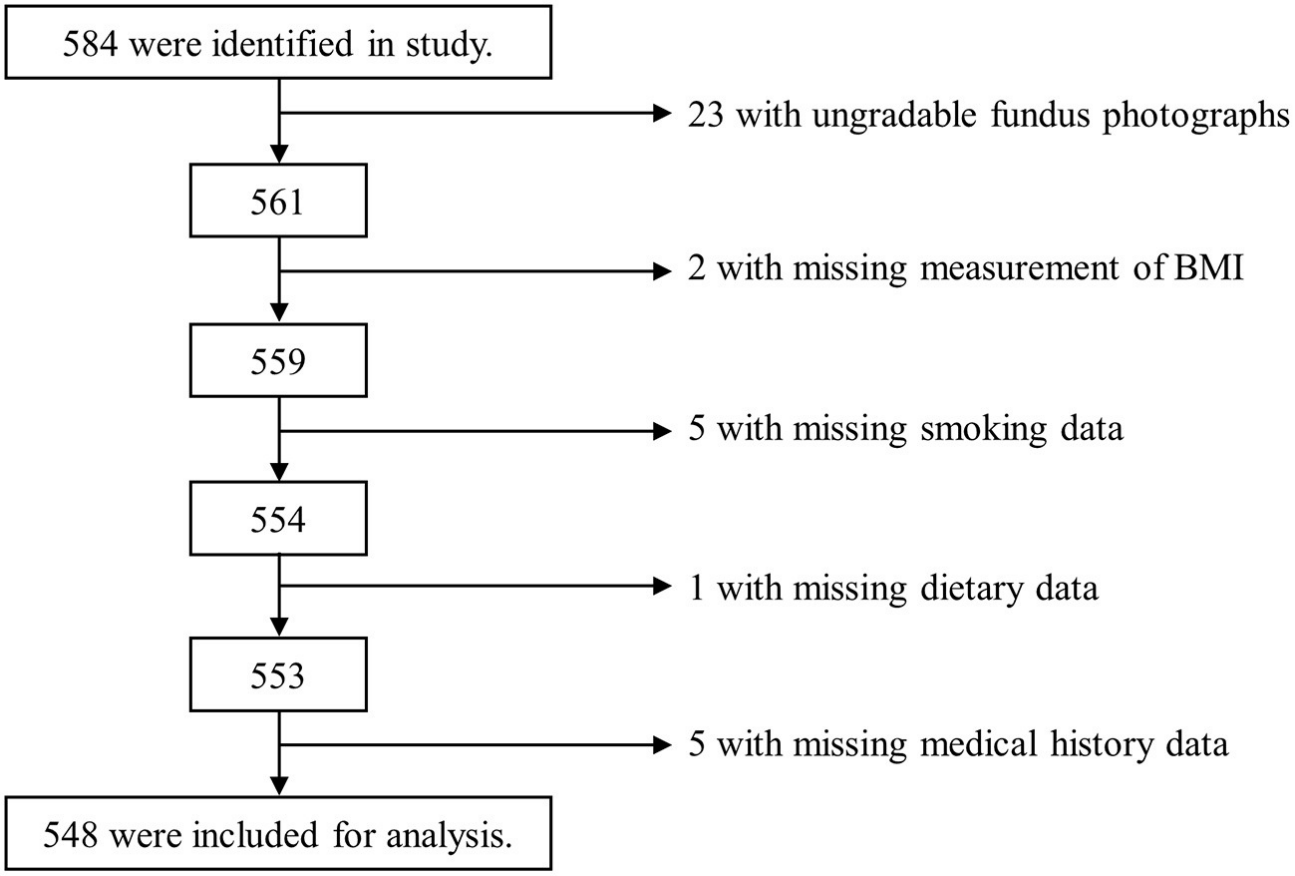
Flow chart showing participant selection in the present study. BMI, body mass index.

Of the 584 participants, we excluded 23 with ungradable fundus photographs and 13 with missing data of BMI, smoking status, dietary intake, and medical history, leaving 548 participants included in the analyses (Figure 1).

Table 1 gives the demographic characteristics of the included participants and mean retinal vessel caliber. Of the overall participants, 38.3% were men and the mean age ± SD was 61.7 ± 13.3 years. The mean CRAE and CRVE was 131.8 ± 15.5 μm and 192.0 ± 19.8 μm, respectively. In univariate analysis, narrower CRAE was related to older age, male sex, hypertension, previous stroke, and obesity. Wider CRVE was related to younger age and current smoking (Table 2). The mean nutrient intake for each quartile is shown in Table 3.

**Table 1:** Demographic characteristics of study participants^a^.

**Characteristics**	***N* = 548**
Age, mean ± SD, years	61.7 ± 13.3
Sex, *N* (%)	
Male	210 (38.3%)
Female	338 (61.7%)
BMI, mean ± SD, kg/m^2^	23.3 ± 3.6
Smoking status, *N* (%)	
Never	324 (59.1%)
Former	161 (29.4%)
Current	63 (11.5%)
Diabetes, *N* (%)	72 (13.1%)
Hypertension, *N* (%)	95 (17.3%)
Dyslipidemia, *N* (%)	323 (58.9%)
HDL-C <40 mg/dL	24 (4.4%)
LDL-C ≥140 mg/dL	181 (33.0%)
TG ≥150 mg/dL	139 (25.3%)
Previous CHD, *N* (%)	13 (2.4%)
Previous stroke, *N* (%)	9 (1.6%)
CRAE, mean ± SD, μm	131.8 ± 15.5
CRVE, mean ± SD, μ m	192.0 ± 19.8

a *Data are presented as number (percentile) unless otherwise indicated. SD, standard deviation; N, number; BMI, body mass index; HDL-C, high-density lipoprotein cholesterol; LDL-C, low-density lipoprotein cholesterol; TG, triglyceride; CHD, coronary heart disease; CRAE, central retinal artery equivalent; CRVE, central retinal vein equivalent*.

**Table 2:** Relationship between participants' background data and retinal vascular caliber (CRAE and CRVE).

		**CRAE**		**CRVE**	
**Variables**	***N***	**Mean ±SD**	***P*-value^a^**	**Mean ±SD**	***P*-value^a^**
Age, years			<0.01		<0.01
<65	284	134.5 ± 14.8		195.3 ± 18.9	
≥65	264	129.0 ± 15.8		188.5 ± 20.2	
Sex			<0.01		0.31
Male	210	129.3 ± 16.6		192.8 ± 20.3	
Female	338	133.4 ± 14.6		191.5 ± 19.5	
BMI, kg/m^2^			<0.01		0.08
<18.5	49	139.7 ± 17.5		195.6 ± 18.3	
18.5–24.9	340	132.0 ± 15.0		190.4 ± 20.1	
≥25	159	129.0 ± 15.2		194.3 ± 19.4	
Smoking status			0.16		<0.01
Never	324	132.2 ± 15.7		190.5 ± 19.5	
Former	161	130.0 ± 15.9		191.4 ± 19.8	
Current	63	135.0 ± 13.0		201.0 ± 19.6	
Hypertension			<0.01		0.16
Yes	95	125.1 ± 14.9		189.6 ± 19.3	
No	453	133.3 ± 15.3		192.5 ± 19.9	
Diabetes			0.35		0.39
Yes	72	130.5 ± 14.8		194.0 ± 22.3	
No	476	132.0 ± 15.6		191.7 ± 19.4	
Dyslipidemia			0.28		0.38
Yes	323	131.5 ± 15.4		192.5 ± 19.3	
No	225	132.3 ± 15.7		191.2 ± 20.5	
HDL-C <40 mg/dL			0.53		0.53
Yes	24	128.5 ± 19.1		195.5 ± 19.2	
No	524	132.0 ± 15.3		191.8 ± 19.8	
LDL-C ≥140 mg/dL			0.27		0.13
Yes	181	133.4 ± 15.2		194.0 ± 19.0	
No	367	131.0 ± 15.6		191.0 ± 20.2	
TG ≥150 mg/dL			0.71		0.06
Yes	139	131.2 ± 15.2		194.7 ± 20.0	
No	409	1 for 32.0 ± 15.6		191.1 ± 19.7	
Previous CHD			0.32		0.88
Yes	13	129.5 ± 6.4		190.5 ± 21.1	
No	535	131.9 ± 15.7		192.0 ± 19.8	
Previous stroke			0.04		0.65
Yes	9	121.9 ± 12.4		190.1 ± 17.4	
No	539	132.0 ± 15.5		192.0 ± 19.9	

a *Analyzed with Wilcoxon rank-sum test to compare two groups (age, sex, hypertension, diabetes, dyslipidemia, HDL-C <40 mg/dL, LDL-C ≥140 mg/dL, TG ≥150 mg/dL, previous CHD and previous stroke) and Kruskal–Wallis test to compare three groups (smoking status and BMI). P < 0.05 was considered significant. SD, standard deviation; CRAE, central retinal artery equivalent; CRVE, central retinal vein equivalent; BMI, body mass index; HDL-C, high-density lipoprotein cholesterol; LDL-C, low-density lipoprotein cholesterol; TG, triglyceride; CHD, coronary heart disease*.

**Table 3:** Mean daily energy-adjusted nutrient intake, by quartile.

**Nutrient**	**Q1**	**Q2**	**Q3**	**Q4**
Animal protein, g	25.0 ± 4.6	33.5 ± 1.8	39.5 ± 1.6	51.9 ± 8.0
Vegetable protein, g	26.2 ± 4.2	32.9 ± 1.2	37.4 ± 1.3	44.2 ± 3.7
Animal fat (g)	9.9 ± 11.8	28.1 ± 3.2	39.0 ± 3.9	72.7 ± 30.8
Vegetable fat (g)	25.1 ± 4.6	32.9 ±1.5	37.9 ± 1.4	46.6 ± 5.4
SFA, g	12.4 ± 3.9	18.8 ± 1.0	22.5 ± 1.1	31.7 ± 7.7
PUFA, g	10.8 ± 1.3	13.3 ± 0.4	14.8 ± 0.6	18.2 ± 2.3
Cholesterol, mg	179.7 ± 37.3	244.0 ± 13.5	290.4 ± 16.0	400.6 ± 84.3
Carbohydrates, g	216.8 ± 55.5	282.0 ± 8.4	308.6 ± 8.4	357.4 ± 32.0
Fiber, g	10.7 ± 1.9	14.1 ± 0.8	17.2 ± 1.0	24.0 ± 4.2
Vitamin A, μgRAE	341.4 ± 79.2	466.6 ± 30.2	644.0 ± 62.6	961.9 ± 207.7
Vitamin B1, mg	0.6 ± 0.1	0.8 ± 0.04	1.0 ± 0.06	1.3 ± 0.2
Vitamin B2, mg	0.8 ± 0.1	1.1 ± 0.06	1.3 ± 0.07	1.7 ± 0.2
Vitamin C, mg	95.1 ± 21.8	139.1 ± 10.9	180.9 ± 15.1	267.0 ± 51.5
Calcium, mg	384.9 ± 63.5	537.7 ± 36.8	661.0 ± 36.2	858.7 ± 99.4
Iron, mg	4.7 ± 0.6	6.1 ± 0.38	7.4 ± 0.4	9.8 ±1.3
Potassium, mg	2,151.8 ± 250.3	2,613.0 ± 111.4	3,088.5 ± 151.3	3,978.5 ± 517.1
Salt, g	2.9 ± 1.1	4.7 ± 0.4	6.3 ± 0.5	8.8 ± 1.1

*SD, standard deviation; SFA, saturated fatty acids; PUFA, polyunsaturated fatty acids; RAE, retinol activity equivalent; Q, quartile*.

### Association of Nutrient Intake With Retinal Vessel Diameters

Table 4 shows the association of dietary nutrient intake with retinal arterial caliber in multivariate linear regression analysis. There was no significant linear association between CRAE and intake of animal protein, vegetable protein, animal fat, vegetable fat, SFA, PUFA, cholesterol, carbohydrates, fiber, vitamins A, B1, B2 and C, calcium, iron, potassium and salt in both model 1 (adjusted for age and sex) and model 2 (adjusted for age, sex, smoking status, BMI, hypertension, diabetes, dyslipidemia, history of CHD, and history of stroke).

**Table 4:** Mean CRAE (μm) differences across quartiles of energy-adjusted nutrient intake compared with the lowest quartiles.

**Nutrient**	**Model**	**Q2 vs. Q1**	**Q3 vs. Q1**	**Q4 vs. Q1**	***P* for trend**
Animal protein	1	4.61 (1.09, 8.14)	2.07 (−1.46, 5.59)	1.61 (−1.92, 5.13)	0.45
	2	4.41 (0.93, 7.89)	1.50 (−2.02, 5.01)	1.93 (−1.59, 5.45)	0.38
Vegetable protein	1	2.46 (−1.08, 6.00)	−1.85 (−5.41, 1.70)	−0.97 (−4.54, 2.60)	0.30
	2	2.14 (−1.37, 5.66)	−2.22 (−5.73, 1.29)	−0.88 (−4.40, 2.65)	0.38
Animal fat	1	1.09 (−2.48, 4.67)	2.32 (−1.28, 5.91)	1.99 (−1.59, 5.57)	0.32
	2	0.32 (−3.24, 3.87)	1.36 (−2.24, 4.95)	1.56 (−1.99, 5.11)	0.41
Vegetable fat	1	0.50 (−3.07, 4.08)	−1.83 (−5.43, 1.76)	0.95 (−2.59, 4.50)	0.98
	2	0.64 (−2.89, 4.17)	−1.41 (−4.99, 2.17)	1.08 (−2.42, 4.59)	0.85
SFA	1	2.65 (−0.95, 6.25)	2.52 (−1.13, 6.17)	2.68 (−0.91, 6.26)	0.20
	2	1.88 (−1.69, 5.46)	1.75 (−1.89, 5.39)	2.31 (−1.27, 5.89)	0.24
PUFA	1	0.28 (−3.28, 3.83)	−0.05 (−3.67, 3.57)	0.69 (−2.86, 4.24)	0.73
	2	0.43 (−3.08, 3.94)	0.18 (−3.42, 3.79)	0.78 (−2.74, 4.30)	0.53
Cholesterol	1	1.08 (−2.47, 4.63)	1.29 (−2.26, 4.83)	1.73 (−1.81, 5.27)	0.16
	2	1.08 (−2.43, 4.58)	0.76 (−2.74, 4.27)	2.29 (−1.22, 5.81)	0.15
Carbohydrates	1	0.98 (−2.56, 4.52)	2.31 (−1.23, 5.85)	0.15 (−3.39, 3.70)	0.97
	2	0.76 (−2.77, 4.28)	2.31 (−1.19, 5.82)	0.58 (−2.94, 4.11)	0.90
Fiber	1	1.16 (−2.40, 4.72)	1.62 (−1.93, 5.18)	−0.15 (−3.70, 3.41)	0.35
	2	0.70 (−2.83, 4.24)	1.22 (−2.31, 4.75)	−0.02 (−3.53, 3.48)	0.62
Vitamin A	1	1.15 (−2.41, 4.70)	1.05 (−2.52, 4.62)	0.14 (−3.42, 3.71)	0.44
	2	0.68 (−2.87, 4.23)	1.00 (−2.57, 4.56)	0.36 (−3.17, −3.89)	0.62
Vitamin B1	1	−0.36 (−3.86, 3.13)	2.26 (−1.26, 5.79)	−0.75 (−4.29, 2.79)	0.73
	2	−0.23 (−3.70, 3.24)	2.31 (−1.24, 5.85)	0.36 (−3.17, 3.89)	0.73
Vitamin B2	1	0.63 (−2.90, 4.16)	1.60 (−1.95, 5.14)	1.27 (−2.24, 4.79)	0.80
	2	0.52 (−2.96, 4.01)	1.89 (−1.62, 5.41)	1.35 (−2.17, 4.88)	0.74
Vitamin C	1	−2.22 (−5.78, 1.35)	0.63 (−2.95, 4.22)	−2.87 (−6.43, 0.70)	0.28
	2	−2.10 (−5.67, 1.48)	0.61 (−2.96, 4.18)	−2.41 (−5.95, 1.13)	0.43
Calcium	1	1.69 (−1.89, 5.26)	1.61 (−2.00, 5.22)	1.20 (−2.41, 4.81)	0.82
	2	1.80 (−1.72, 5.32)	2.22 (−1.35, 5.79)	1.31 (−2.26, 4.88)	0.69
Iron	1	0.84 (−2.71, 4.39)	−0.66 (−4.21, 2.89)	−1.61 (−5.15, 1.93)	0.24
	2	0.38 (−3.14, 3.90)	−0.76 (−4.29, 2.76)	−1.33 (−4.83, 2.17)	0.34
Potassium	1	−0.52 (−4.08, 3.04)	−0.95 (−4.53, 2.63)	−1.02 (−4.59, 2.55)	0.32
	2	−1.12 (−4.68, 2.44)	−1.30 (−4.89, 2.29)	−0.81 (−4.35, 2.72)	0.45
Salt	1	−2.33 (−5.87, 1.22)	−3.11 (−6.64, 0.43)	−0.87 (−4.43, 2.68)	1.00
	2	−2.05 (−5.58, 1.47)	−2.88 (−6.39, 0.64)	−0.63 (−4.17, 2.90)	0.95

*The 95% confidence interval is in parentheses. Model 1: adjusted for age and sex. Model 2: adjusted for age, sex, smoking status, body mass index, hypertension, diabetes, dyslipidemia, history of coronary heart disease and stroke. CRAE, central retinal artery equivalent; Q, quartile; SFA, saturated fatty acids; PUFA, polyunsaturated fatty acids*.

Table 5 demonstrates the association of nutrient intake in multivariate linear regression analysis for retinal venular caliber. After adjustment for age and sex (model 1), there were significant inverse associations of retinal venular caliber with vitamins A, C, and potassium [mean difference for the second, third, and highest quartiles: −2.35 (95% CI: −6.94, 2.24), −2.27 (−6.88, 2.33), and −5.70 (−10.29 to −1.11), *P*-value for trend = 0.01 for vitamin A; −2.12 (−6.74, 2.50), −0.39 (−5.04, 4.25), and −5.20 (−9.82 to −0.58), *P*-value for trend = 0.02 for vitamin C; −2.67 (−7.27, 1.94), −4.61 (−9.23, 0.02), and −3.95 (−8.56, 0.66), *P*-value for trend = 0.04 for potassium]. We found that higher intake of vitamins A, C and potassium had a significant inverse association with venular caliber after adjusting for age, sex, smoking status, BMI, hypertension, diabetes, dyslipidemia and history of diagnosed CHD and stroke (model 2); mean difference for the second, third, and highest quartiles: −2.55 (95% CI: −7.15, 2.05), −1.83 (−6.45, 2.78), and −5.33 (−9.91 to −0.76), *P*-value for trend = 0.02 for vitamin A; −2.18 (−6.83, 2.47), −0.27 (−4.92, 4.37), and −4.93 (−9.54 to −0.32), *P*-value for trend = 0.02 for vitamin C; −2.64 (−7.26, 1.97), −4.58 (−9.24, 0.07), −3.90 (−8.48, 0.69), *P*-value for trend = 0.04 for potassium. There were no significant associations of CRVE with animal protein, vegetable protein, animal fat, vegetable fat, SFA, PUFA, cholesterol, carbohydrates, fiber, vitamin B1, vitamin B2, calcium, iron, or salt in either model.

**Table 5:** Mean CRVE (μm) differences across quartiles of energy-adjusted nutrient intake compared with the lowest quartiles.

**Nutrient**	**Model**	**Q2 vs. Q1**	**Q3 vs. Q1**	**Q4 vs. Q1**	***P* for trend^a^**
Animal protein	1	2.86 (−1.73, 7.45)	3.48 (−1.11, 8.06)	1.33 (−159, 5.91)	0.84
	2	2.53 (−2.02, 7.07)	1.91 (−2.69, 6.50)	0.27 (−4.33, 4.88)	0.80
Vegetable protein	1	−0.09 (−4.70, 4.52)	−0.96 (−5.59, 3.67)	−2.41 (−7.06, 2.24)	0.24
	2	−0.22 (−4.82, 4.38)	−0.56 (−5.16, 4.03)	−2.40 (−7.01, 2.21)	0.29
Animal fat	1	0.89 (−3.74, 5.52)	3.30 (−1.36, 7.96)	0.69 (−3.95, 5.32)	0.95
	2	0.22 (−4.40, 4.04)	3.06 (−1.61, 7.73)	0.51 (−4.11, 5.13)	0.95
Vegetable fat	1	−1.92 (−6.56, 2.73)	−2.41 (−7.08, 2.26)	−0.72 (−5.32, 3.89)	0.45
	2	−2.35 (−6.94, 2.25)	−3.00 (−7.66, 1.67)	−1.40(−5.97, 3.16)	0.28
SFA	1	0.60 (−4.07, 5.28)	1.99 (−2.75, 6.73)	−0.03 (−4.69, 4.63)	0.95
	2	0.55 (−4.10, 5.21)	1.72 (−3.02, 6.46)	−0.08 (−4.75, 4.58)	0.90
PUFA	1	0.41 (−4.20, 5.01)	−0.57 (−5.26, 4.12)	−0.17 (−4.78, 4.43)	0.38
	2	0.23 (−4.34, 4.80)	−1.48 (−6.17, 3.21)	−1.19 (−5.77, 3.39)	0.21
Cholesterol	1	−0.34 (−4.93, 4.25)	3.33 (−1.24, 7.91)	−1.08 (−5.66, 3.49)	0.52
	2	−0.43 (−4.98, 4.12)	3.38 (−1.18, 7.93)	−0.97 (−5.53, 3.59)	0.59
Carbohydrates	1	3.56 (−1.03, 8.14)	2.49 (−2.09, 7.07)	0.50 (−4.08, 5.09)	0.56
	2	3.34 (−1.25, 7.92)	2.45 (−2.10, 7.01)	0.92 (−3.67, 5.51)	0.46
Fiber	1	−1.55 (−6.17, 3.06)	−0.49 (−5.10, 4.12)	−4.82 (−9.44, −0.19)	0.05
	2	−0.57 (−5.16, 4.02)	0.33 (−4.25, 4.91)	−3.37 (−7.92, 1.19)	0.06
Vitamin A	1	−2.35 (−6.94, 2.24)	−2.27 (−6.88, 2.33)	−5.70 (−10.29, −1.11)	0.01
	2	−2.55 (−7.15, 2.05)	−1.83 (−6.45, 2.78)	−5.33 (−9.91, −0.76)	0.02
Vitamin B1	1	0.37 (−4.15, 4.89)	3.91 (−0.65, 8.48)	−1.23 (−5.81, 3.34)	0.88
	2	−0.76 (−5.27, 3.76)	2.39 (−2.215, 7.00)	−2.58 (−7.18, 2.01)	0.42
Vitamin B2	1	−0.67 (−5.25, 3.91)	0.51 (−4.08, 5.11)	−1.17 (−5.74, 3.39)	0.69
	2	−1.06 (−5.60, 3.48)	0.17 (−4.41, 4.75)	−2.13 (−6.72, 2.46)	0.44
Vitamin C	1	−2.12 (−6.74, 2.50)	−0.39 (−5.04, 4.25)	−5.20 (−9.82, −0.58)	0.02
	2	−2.18 (−6.83, 2.47)	−0.27 (−4.92, 4.37)	−4.93 (−9.54, −0.32)	0.02
Calcium	1	−1.68 (−6.30, 2.94)	−4.91 (−9.57, −0.24)	−2.41 (−7.07, 2.26)	0.40
	2	−1.02 (−5.60, 3.55)	−4.40 (−9.04, 0.24)	−2.61 (−7.25, 2.03)	0.31
Iron	1	1.46 (−3.13, 6.04)	−2.38 (−6.97, 2.21)	−3.38 (−7.96, 1.20)	0.05
	2	1.60 (−2.97, 6.16)	−2.63 (−7.19, 1.94)	−3.04 (−7.58, 1.49)	0.05
Potassium	1	−2.67 (−7.27, 1.94)	−4.61 (−9.23, 0.02)	−3.95 (−8.56, 0.66)	0.04
	2	−2.64 (−7.26, 1.97)	−4.58 (−9.24, 0.07)	−3.90 (−8.48, 0.69)	0.04
Salt	1	−0.38 (−4.98, 4.22)	2.12 (−2.47, 6.70)	−2.07 (−6.68, 2.55)	0.52
	2	−0.64 (−5.23, 3.94)	1.87 (−2.71, 6.44)	−2.62 (−7.21, 1.98)	0.35

*The 95% confidence interval is in parentheses. Model 1: adjusted for age and sex. Model 2: adjusted for age, sex, smoking status, body mass index, hypertension, diabetes, dyslipidemia, history of coronary heart disease and stroke*.

a *P < 0.05 considered significant. CRVE, central retinal vein equivalent; Q, quartile; SFA, saturated fatty acids; PUFA, polyunsaturated fatty acids*.

**Figure 2:**
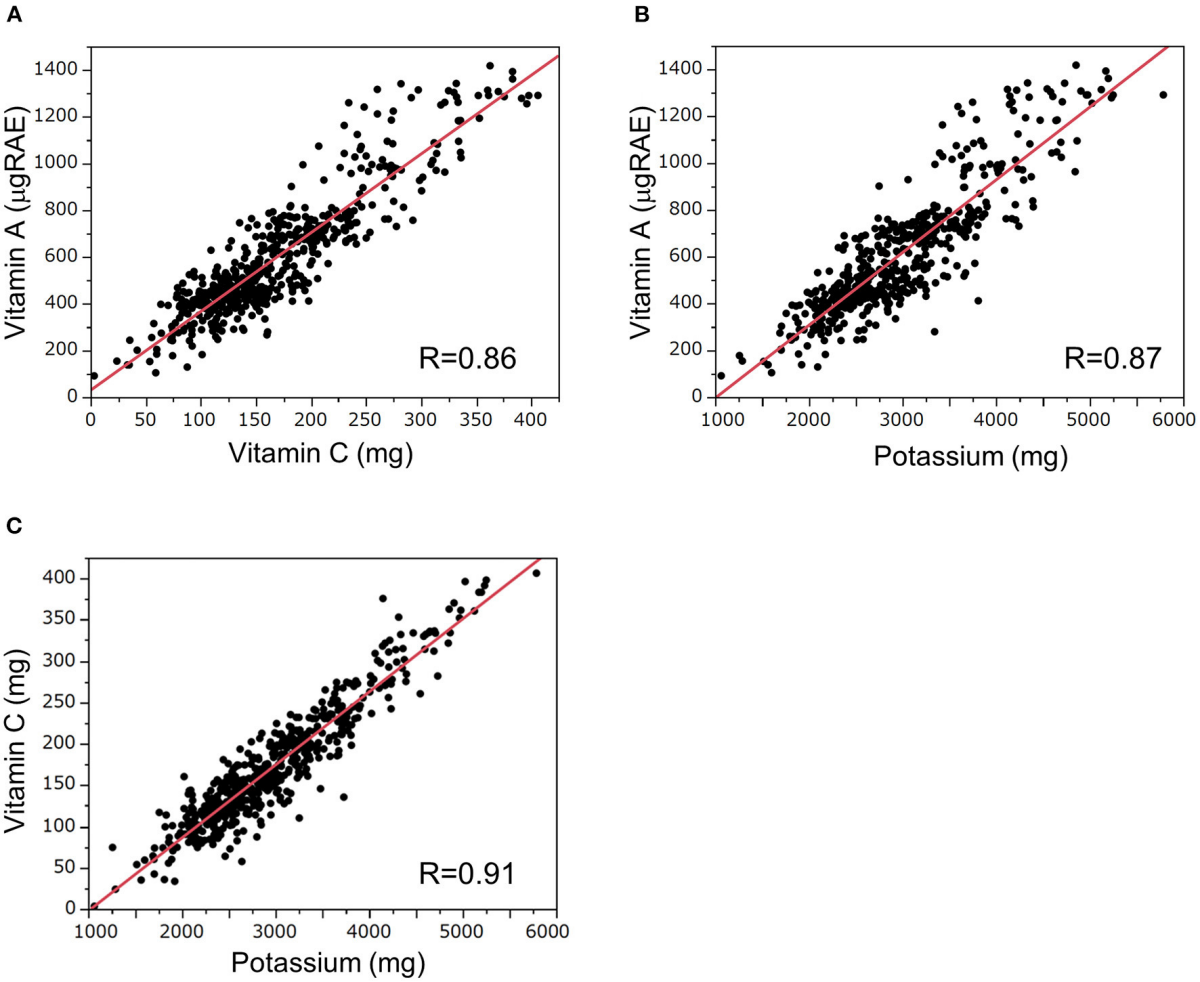
Correlations of dietary daily intakes of vitamins A, C, and potassium. Correlations between dietary intake of **(A)** vitamin A and vitamin C, **(B)** vitamin A and potassium, and **(C)** vitamin C and potassium. The correlations were analyzed by Spearman's rank correlation test. RAE, retinol activity equivalent.

Correlations between the intake of these nutrients (vitamins A, C, and potassium) are shown in Figure 2. Intake of vitamin A and vitamin C, vitamin A and potassium, and vitamin C and potassium were significantly correlated with each other (vitamin A vs. vitamin C, *R* = 0.86, *P* < 0.01; vitamin A vs. potassium, *R* = 0.87, *P* < 0.01; vitamin C vs. potassium, *R* = 0.91, *P* < 0.01).

## Discussion

In the present study, we found that vitamins A, C and potassium intake had an inverse association with retinal venular caliber after accounting for cardiovascular disease factors. The nutritional intake of the population in this study generally did not greatly deviate from the U.S. Dietary Reference Intakes (33). With respect to vitamin C, the recommended intake of vitamin C is 75–90 mg/day (33); the majority of participants in this study met the recommended intake. Previous studies have shown that intake of vitamins A and C, which have antioxidant properties, contributes to a reduction in the risk of CHD and stroke (10, 12, 14). Adequate dietary potassium reduces the risk of CHD and stroke (34). Retinal vessel diameter, which reflects systemic microvascular status, has been shown to be associated with CHD and stroke outcomes, and epidemiological studies have shown that wider CRVE is independently associated with future risk of CHD and stroke (1, 5, 6). Although the association between these individual nutrient intake and the structure of the retinal microvasculature has not been reported, our results are supported by Gopinath et al. (15). They reported that a high-quality diet rich in fruits and vegetables was associated with narrower retinal venular caliber, indicating better retinal microvascular health. Vitamins A, C and potassium come largely from fruits and vegetables (34), and our results are consistent with their report.

We consider that the antioxidant effects of vitamins A and C might be involved in our finding that higher intake of vitamins A and C prevented retinal venular caliber enlargement. It has been suggested that retinal venular caliber widening underlies destruction of the endothelial surface layer (ESL) (35). Indeed, Wong et al. reported that wider retinal venular caliber is related to higher levels of soluble intercellular adhesion molecule-1 and plasminogen activator inhibitor-1, biomarkers of endothelial dysfunction (26). Tamai et al. reported that oxidative stress caused by lipid hydroperoxide injection into the vitreous of rats resulted in an increase in the number of leukocytes in the retinal microvasculature and an enlarged retinal venular caliber (36). Epidemiological studies have also reported that systemic inflammatory markers such as white blood cell count, erythrocyte sedimentation rate, high-sensitivity c-reactive protein (CRP), interleukin-6, and serum amyloid A are associated with wide retinal venular caliber (35, 37).

Antioxidants, such as vitamins A and C, are considered to protect against oxidant-mediated inflammation by virtue of their capacity to scavenge reactive oxygen species (ROS) and inhibit the activation of nuclear factor kappa-B (NF-κB), a transcription factor that promotes the expression of genes that induce inflammation (38, 39). Previous studies have shown that blood concentrations of vitamins A and C have a negative association with CRP, a marker of inflammation (40–42). Additionally, Hermersson et al. showed that higher dietary β-carotene and vitamin C intake significantly reduced formation of F2-isoprostanes, a marker of oxidative stress (43). Experimental studies have also reported that vitamins A and C inhibit the activation of NF-κB, which regulates the promotion of inflammation (44, 45). The detailed mechanism by which antioxidants prevent the enlargement of retinal venular caliber remains to be elucidated. However, according to the evidence presented above, we consider that higher intake of vitamins A and C prevented ESL destruction by reducing oxidative stress and inhibiting the development of inflammation, thereby preventing retinal venular caliber widening.

Hypertension is a risk factor for CHD and stroke. It has been considered that the antihypertensive effect of potassium is responsible for the reduced risks of CHD and stroke associated with high potassium intake (46). However, Tobian et al. reported that the incidences of stroke and death were drastically reduced in rats with high potassium intake, regardless of identical blood pressure (47). These findings suggest that potassium has a beneficial effect on blood vessels through mechanisms other than its antihypertensive effect. The mechanism has not yet been fully elucidated; however, the antioxidant effect may be a contributing factor. Although vitamins A and C are the typical antioxidant nutrients, a potassium diet also has antioxidant effect, and it reduces the free radical formation (34). He et al. showed that without changing blood pressure, 64 mmol of potassium, whether as the chloride or bicarbonate salt, improved vascular endothelial function in 42 adults (48). Our result might be explained by the antioxidant effects described above. However, in this study, there were significant correlations between the intake of vitamins A, C and potassium. Vitamins A, C, and potassium are abundant in fruits and vegetables; therefore, it might not be possible to simply conclude that high potassium intake is related to narrower CRVE.

In this study, no significant association was found between dietary nutrient intake and retinal arterial caliber. In particular, there was no significant association of arterial caliber with vitamins A, C and potassium which were found to be associated with venous caliber. In previous animal studies, administration of lipid hydroperoxide did not result in narrowing of the retinal arterial caliber (36), which is consistent with our results. Although the mechanism explaining this observation is unclear, these findings suggest that ROS/antioxidants might only affect the retinal venous caliber and not the arterioles. As there are still few reports on the relationship between antioxidants and retinal blood vessel diameter, it is possible that confounding factors not analyzed in this study may have influenced the results. Both arterial caliber narrowing and venous caliber enlargement have been considered to indicate deterioration of the systemic microvascular circulation; however, in recent years, the role of venules, independent of arterioles, has been attracting attention. In particular, wider venular caliber has been shown to be a potentially important marker of micro-vascular disease (35). Smoking has also been shown to be associated with only a wider venular caliber but not arterial caliber (1, 49). Further research is needed to fully elucidate all aspects of the effects of nutrient intake on the microcirculatory system.

There are several limitations in this study. First, the number of participants was limited. Second, the participants in this study were not representative of the general population. Hence, our results might not be relevant to the general population as the current study only included Japanese American individuals. Third, our study used a cross-sectional design; thus, it was impossible to determine the directional associations. Fourth, physical activity, socioeconomic factors, eye condition, and serum levels of vitamins and minerals were not examined. In myopic eyes, retinal vessel caliber is reportedly narrower because of longer ocular axis (50). To clarify the causal relationships between vitamins A, C, and potassium with the microvasculature, prospective studies with larger sample sizes drawn from the general population are needed; such studies should examine serum levels of vitamins and minerals, ocular conditions (e.g., refractive status and ocular axial length), and physical and social factors.

In conclusion, we showed that vitamins A, C and potassium intake was inversely associated with retinal venular caliber. This suggests that dietary intake of vitamins A, C and potassium might be beneficial for a healthy retinal microvascular profile. The retinal vasculature provides a non-invasive window into the status of systemic microvascular (1). This study provides insights for clarifying the effects of dietary nutrition on microvasculature. We would like to emphasize that this is the first time that the association of dietary vitamins A, C and potassium intake with retinal vascular status has been demonstrated; therefore, further prospective studies are needed to assess whether the evidence is consistent.

## Data Availability Statement

The data analyzed in this study are available from the corresponding author on reasonable request.

## Ethics Statement

The studies involving human participants were reviewed and approved by Institutional Review Board of Hiroshima University (approval No. E-139). The participants provided their written informed consent to participate in this study.

## Author Contributions

YK: conceptualization. HO and MY: data curation. AE: formal analysis. DI, RT, MK, AN, and HO: investigation. AE and DI: writing—original draft preparation. KH, RK, MY, and YK: writing—review and editing. KH, MY, and YK: supervision. All authors contributed to the article and approved the submitted version.

## Funding

This study was financially supported by Japan Society for the Promotion of Science KAKENHI Grant (No. JP16K09035) to MY.

## Conflict of Interest

The authors declare that the research was conducted in the absence of any commercial or financial relationships that could be construed as a potential conflict of interest.

## Publisher's Note

All claims expressed in this article are solely those of the authors and do not necessarily represent those of their affiliated organizations, or those of the publisher, the editors and the reviewers. Any product that may be evaluated in this article, or claim that may be made by its manufacturer, is not guaranteed or endorsed by the publisher.

## References

[1] Sun C Wang JJ Mackey DA Wong TY. Retinal vascular caliber: systemic, environmental, and genetic associations. Surv Ophthalmol. (2009) 54:74–95. 10.1016/j.survophthal.2008.10.003 19171211

[2] Serre K Sasongko MB. Modifiable lifestyle and environmental risk factors affecting the retinal microcirculation. Microcirculation. (2012) 19:29–36. 10.1111/j.1549-8719.2011.00121.x 21740476

[3] McEvoy CT Wallace IR Hamill LL Neville CE Hunter SJ Patterson CC. Increasing fruit and vegetable intake has no effect on retinal vessel caliber in adults at high risk of developing cardiovascular disease. Nutr Metab Cardiovasc Dis. (2016) 26:318–25. 10.1016/j.numecd.2015.10.010 27004617

[4] Wong TY Knudtson MD Klein R Klein BEK Meuer SM Hubbard LD. Computer-assisted measurement of retinal vessel diameters in the beaver dam eye study: methodology, correlation between eyes, and effect of refractive errors. Ophthalmology. (2004) 111:1183–90. 10.1016/j.ophtha.2003.09.039 15177969

[5] McGeechan K Liew G Macaskill P Irwig L Klein R Klein BEK. Prediction of incident stroke events based on retinal vessel caliber: a systematic review and individual-participant meta-analysis. Am J Epidemiol. (2009) 170:1323–32. 10.1093/aje/kwp306 19884126 PMC2800263

[6] McGeechan K Liew G Macaskill P Irwig L Klein R Klein BEK. Meta-analysis: retinal vessel caliber and risk for coronary heart disease. Ann Intern Med. (2009) 151:404. 10.7326/0003-4819-151-6-200909150-00005 19755365 PMC2887687

[7] He FJ Nowson CA Lucas M Macgregor GA. Increased consumption of fruit and vegetables is related to a reduced risk of coronary heart disease: meta-analysis of cohort studies. J Hum Hypertens. (2007) 21:717–28. 10.1038/sj.jhh.1002212 17443205

[8] Leung Yinko SSL Stark KD Thanassoulis G Pilote L. Fish consumption and acute coronary syndrome: a meta-analysis. Am J Med. (2014) 127:848–57.e2. 10.1016/j.amjmed.2014.04.016 24802020

[9] Afshin A Micha R Khatibzadeh S Mozaffarian D. Consumption of nuts and legumes and risk of incident ischemic heart disease, stroke, and diabetes: a systematic review and meta-analysis. Am J Clin Nutr. (2014) 100:278–88. 10.3945/ajcn.113.076901 24898241 PMC4144102

[10] Gaziano JM Manson JE Branch LG Colditz GA Willett WC Buring JE. A prospective study of consumption of carotenoids in fruits and vegetables and decreased cardiovascular mortality in the elderly. Ann Epidemiol. (1995) 5:255–60. 10.1016/1047-2797(94)00090-g 8520706

[11] Ascherio A Rimm EB HernáN MA Giovannucci EL Kawachi I Stampfer MJ. Intake of potassium, magnesium, calcium, and fiber and risk of stroke among US men. Circulation. (1998) 98:1198–204. 10.1161/01.cir.98.12.1198 9743511

[12] Vokó Z Hollander M Hofman A Koudstaal PJ Breteler MM. Dietary antioxidants and the risk of ischemic stroke: the rotterdam study. Neurology. (2003) 61:1273–5. 10.1212/01.wnl.0000090458.67821.a3 14610137

[13] Nakanishi S Okubo M Yoneda M Jitsuiki K Yamane K Kohno N. A comparison between Japanese-Americans living in Hawaii and los angeles and native Japanese: the impact of lifestyle westernization on diabetes mellitus. Biomed Pharmacother. (2004) 58:571–7. 10.1016/j.biopha.2004.10.001 15589065

[14] Kubota Y Iso H Date C Kikuchi S Watanabe Y Wada Y. Dietary intakes of antioxidant vitamins and mortality from cardiovascular disease. Stroke. (2011) 42:1665–72. 10.1161/strokeaha.110.601526 21512181

[15] Gopinath B Flood VM Wang JJ Rochtchina E Wong TY Mitchell P. Is quality of diet associated with the microvasculature? An analysis of diet quality and retinal vascular calibre in older adults. Br J Nutr. (2013) 110:739–46. 10.1017/s0007114512005491 23531363

[16] Keel S Itsiopoulos C Koklanis K Vukicevic M Cameron F Gilbertson H. Dietary patterns and retinal vascular calibre in children and adolescents with type 1 diabetes. Acta Ophthalmol. (2016) 94:e345–52. 10.1111/aos.12941 26749006

[17] Kan H Stevens J Heiss G Klein R Rose KM London SJ. Dietary fiber intake and retinal vascular caliber in the atherosclerosis risk in communities study. Am J Clin Nutr. (2007) 86:1626–32. 10.1093/ajcn/86.5.1626 18065579 PMC2190622

[18] Gopinath B Flood VM Burlutsky G Louie JCY Baur LA Mitchell P. Dairy food consumption, blood pressure and retinal microcirculation in adolescents. Nutr Metab Cardiovasc Dis. (2014) 24:1221–7. 10.1016/j.numecd.2014.05.014 24996501

[19] Kaushik S Wang JJ Wong TY Flood V Barclay A Brand-Miller J. Glycemic index, retinal vascular caliber, and stroke mortality. Stroke. (2009) 40:206–12. 10.1161/strokeaha.108.513812 18948616

[20] Yoneda M Kobuke K. A 50-year history of the health impacts of westernization on the lifestyle of Japanese Americans: a focus on the hawaii–los angeles–hiroshima study. J Diabetes Investig. (2020) 11:1382–7. 10.1111/jdi.13278 32311224 PMC7610102

[21] Sugihiro T Yoneda M Ohno H Oki K Hattori N. Associations of nutrient intakes with obesity and diabetes mellitus in the longitudinal medical surveys of Japanese Americans. J Diabetes Investig. (2019) 10:1229–36. 10.1111/jdi.13010 30663246 PMC6717818

[22] Yoserizal M Hirooka K Yoneda M Ohno H Kobuke K Kawano R. Associations of nutrient intakes with glaucoma among Japanese Americans. Medicine (Baltimore). (2019) 98:e18314. 10.1097/md.0000000000018314 31804379 PMC6919431

[23] Edo A Pertiwi YD Hirooka K Masuda S Kamaruddin MI Yanagi M. Association of dietary nutrient intake with early age-related macular degeneration in Japanese-Americans. Metabolites. (2021) 11:673. 10.3390/metabo11100673 34677388 PMC8537321

[24] U.S. Department of Agriculture. The Agricultural Research Service. Nutritive Value of American Foods in Common Units. Washington, DC: Agriculture Handbook (1975).

[25] Hubbard LD Brothers RJ King WN Clegg LX Klein R Cooper LS. Methods for evaluation of retinal microvascular abnormalities associated with hypertension/sclerosis in the atherosclerosis risk in communities study. Ophthalmology. (1999) 106:2269–80. 10.1016/s0161-6420(99)90525-0 10599656

[26] Wong TY Islam FMA Klein R Klein BEK Cotch MF Castro C. Retinal vascular caliber, cardiovascular risk factors, and inflammation: the multi-ethnic study of atherosclerosis (MESA). Invest Ophthalmol Vis Sci. (2006) 47:2341. 10.1167/iovs.05-1539 16723443 PMC2258139

[27] Knudtson MD Lee KE Hubbard LD Wong TY Klein R Klein BEK. Revised formulas for summarizing retinal vessel diameters. Curr Eye Res. (2003) 27:143–9. 10.1076/ceyr.27.3.143.16049 14562179

[28] Kubota M Yoneda M Maeda N Ohno H Oki K Funahashi T. Westernization of lifestyle affects quantitative and qualitative changes in adiponectin. Cardiovasc Diabetol. (2017) 16:83. 10.1186/s12933-017-0565-z 28683803 PMC5501538

[29] Nath Kundu R Biswas S Das M. Mean arterial pressure classification: a better tool for statistical interpretation of blood pressure related risk covariates. Cardiol Angiol. (2017) 6:1–7. 10.9734/ca/2017

[30] American Diabetes Association. Diagnosis and classification of diabetes mellitus. Diabetes Care. (2014) 37(Supple. 1):S81–90. 10.2337/dc14-s081 24357215

[31] Teramoto T Sasaki J Ishibashi S Birou S Daida H Dohi S. Diagnostic criteria for dyslipidemia. Executive summary of the Japan atherosclerosis society (JAS) guidelines for the diagnosis and prevention of Atherosclerotic cardiovascular diseases in Japan-−2012 version. J Atheroscler Thromb. (2013) 20:655–60. 10.5551/jat.15792 23665881

[32] Willett WC Howe GR Kushi LH. Adjustment for total energy intake in epidemiologic studies. Am J Clin Nutr. (1997) 65:1220S−8S. 10.1093/ajcn/65.4.1220s 9094926

[33] Institute Institute of Medicine Committee to Review Dietary Reference Intakes for Vitamin D Calcium. The national academies collection: reports funded by national institutes of health. In: Ross AC Taylor CL Yaktine AL Del Valle HB editors. Dietary Reference Intakes for Calcium Vitamin D. Washington, DC: National Academies Press (US), Copyright © 2011, National Academy of Sciences (2011).

[34] Weaver CM. Potassium and health. Adv Nutr. (2013) 4:368S−77S. 10.3945/an.112.003533 23674806 PMC3650509

[35] Ikram MK De Jong FJ Vingerling JR Witteman JCM Hofman A Breteler MMB. Are retinal arteriolar or venular diameters associated with markers for cardiovascular disorders? The rotterdam study. Invest Ophthalmol Vis Sci. (2004) 45:2129. 10.1167/iovs.03-1390 15223786

[36] Tamai K Matsubara A Tomida K Matsuda Y Morita H Armstrong D. Lipid hydroperoxide stimulates leukocyte–endothelium interaction in the retinal microcirculation. Exp Eye Res. (2002) 75:69–75. 10.1006/exer.2002.1178 12123638

[37] Klein R Klein BEK Knudtson MD Wong TY Tsai MY. Are inflammatory factors related to retinal vessel caliber? Arch Ophthalmol. (2006) 124:87. 10.1001/archopht.124.1.87 16401789

[38] Conner EM Grisham MB. Inflammation, free radicals, and antioxidants. Nutrition. (1996) 12:274–7. 10.1016/s0899-9007(96)00000-8 8862535

[39] Ford ES Liu S Mannino DM Giles WH Smith SJ. C-reactive protein concentration and concentrations of blood vitamins, carotenoids, and selenium among United States adults. Eur J Clin Nutr. (2003) 57:1157–63. 10.1038/sj.ejcn.1601667 12947436

[40] Louw JA Werbeck A Louw ME Kotze TJ Cooper R Labadarios D. Blood vitamin concentrations during the acute-phase response. Crit Care Med. (1992) 20:934–41. 10.1097/00003246-199207000-00007 1617986

[41] Boosalis MG Snowdon DA Tully CL Gross MD. Acute phase response and plasma carotenoid concentrations in older women: findings from the nun study. Nutrition. (1996) 12:475–8. 10.1016/s0899-9007(96)91720-7 8878137

[42] Root MM Hu J Stephenson LS Parker RS Campbell TC. Determinants of plasma retinol concentrations of middle-aged women in rural China. Nutrition. (1999) 15:101–7. 10.1016/s0899-9007(98)00173-7 9990573

[43] Helmersson J Ärnlöv J Larsson A Basu S. Low dietary intake of β-carotene, α-tocopherol and ascorbic acid is associated with increased inflammatory and oxidative stress status in a Swedish cohort. Br J Nutr. (2008) 101:1775–82. 10.1017/s0007114508147377 19079838

[44] Bowie AG O'Neill LAJ. Vitamin C inhibits NF-κB activation by TNF via the activation of p38 mitogen-activated protein kinase. J Immunol. (2000) 165:7180–8. 10.4049/jimmunol.165.12.7180 11120850

[45] Shi HY Yan SM Guo YM Zhang BQ Guo XY Shi BL. Vitamin A pretreatment protects NO-induced bovine mammary epithelial cells from oxidative stress by modulating Nrf2 and NF-κB signaling pathways. J Anim Sci. (2018) 96:1305–16. 10.1093/jas/sky037 29669072 PMC6140872

[46] He FJ MacGregor GA. Fortnightly review: beneficial effects of potassium. BMJ. (2001) 323:497–501. 10.1136/bmj.323.7311.497 11532846 PMC1121081

[47] Tobian L. High-potassium diets markedly protect against stroke deaths and kidney disease in hypertensive rats, an echo from prehistoric days. J Hypertens Suppl. (1986) 4:S67–76. 3464706

[48] He FJ Marciniak M Carney C Markandu ND Anand V Fraser WD. Effects of potassium chloride and potassium bicarbonate on endothelial function, cardiovascular risk factors, and bone turnover in mild hypertensives. Hypertension. (2010) 55:681–8. 10.1161/hypertensionaha.109.147488 20083724

[49] Yanagi M Misumi M Kawasaki R Takahashi I Itakura K Fujiwara S. Is the association between smoking and the retinal venular diameter reversible following smoking cessation? Invest Ophthalmol Vis Sci. (2014) 55:405. 10.1167/iovs.13-12512 24302587

[50] Li H Mitchell P Rochtchina E Burlutsky G Wong TY Wang JJ. Retinal vessel caliber and myopic retinopathy: the blue mountains eye study. Ophthalmic Epidemiol. (2011) 18:275–80. 10.3109/09286586.2011.602508 22053837

